# Factors that affect immunization data quality in Kabarole District, Uganda

**DOI:** 10.1371/journal.pone.0203747

**Published:** 2018-09-21

**Authors:** Fred Nsubuga, Henry Luzze, Immaculate Ampeire, Simon Kasasa, Opar Bernard Toliva, Alex Ario Riolexus

**Affiliations:** 1 Public Health Fellowship Program, Kampala, Uganda; 2 Uganda National Expanded Program on Immunization, Ministry of Health, Kampala, Uganda; 3 Makerere University School of Public Health, Kampala, Uganda; Oregon Health and Science University, UNITED STATES

## Abstract

**Introduction:**

Reliable and timely immunization data is vital at all levels of health care to inform decisions and improve program performance. Inadequate data quality may impair our understanding of the true vaccination coverage and also hinder our capability to meet the program objectives. It’s therefore important to regularly assess immunization data quality to ensure good performance, sound decision making and efficient use of resources.

**Methods:**

We conducted an immunization data quality audit between July and August 2016. The verification factor was estimated by dividing the recounted diphtheria, pertussis and tetanus third dose vaccination for children under 1 year (DPT3<1 year) by reported DPT3<1 year. The quality of data collection processes was measured using quality indices for the 3 different components: recording practices, storage/reporting, monitoring and evaluation. These indices were applied to the different levels of the health care service delivery system. Quality index score was estimated by dividing the total question or observation correctly answered by the total number of answers/ observations for a particular component.

**Results:**

The mean health center verification factor was 87%. Sixty five percent (32/49) of the health centers had consistent data, 27% (13/49) over reported and 4% (2/49) under-reported. Health center 11s and 111s contributed to over-reporting and under-reporting. All the health centers’ reports were complete and timely between January and June and from November to December. The mean quality indices for the 3 different componets assessed were; recording practices 66%, storing/reporting 75%, monitoring and evaluation 43%. There was a weak positive correlation between the health center verifaction factor and quality index though this was not statistically significant (r = 0.014; p = 0.92).

**Conclusion:**

Lower level health centers contributed significantly to the inconsistencies in immunization data; there were wide variation between the quality indices of recording practices, storage/reporting, monitoring and evaluation. We recommended that District Local Governments and Ministry of Health focus on improving data quality at lower levels of health service delivery.

## Introduction

Poor data quality and use has been cited by the Global Vaccine Action Plan as one of the five key priorities that need to be addressed, if progress in immunization is to be realized [1].

Data quality is the ability of data to support sound decisions based on statistical inferences [2]. Standards are needed to assess whether the statistics available to decision-makers are comprehensive, timely, accessible and reliable. Inadequate data quality may impair our understanding of the true vaccination coverage and may hinder our capability to meet the program objectives. It’s therefore important to regularly assess data quality to ensure good performance, sound decision making and efficient use of resources. The collection, analysis and use of data to measure and improve immunization program performance have been set as priority areas for country programs and immunization partners [3]. At the inception of Uganda National Expanded Program on Immunization (UNEPI) in 1983, data management was largely paper based. In 1985, Uganda started implementing a health information system to capture morbidity data for selected communicable and non-communicable diseases including immunization [4]. This Health Management Information System (HMIS) model was based on paper tools (reporting forms, registers, databases and manuals) [5]. Due to the many report forms that were supposed to be filled by the health workers, a lot of time was spent in compilation and in the end this was counterproductive. Between 1992 and 1997, HMIS was revised to include management aspects in addition to the existing variables. To further improve on efficiency and effectiveness of the data management system, HMIS was reviewed in 2000 with incorporation of the Health Sector Strategic Plan indicators and was launched nationwide in 2001 [6]. Since then, there has been revisions every 5 years. In 2012, the electronic version of the health management information system (eHMIS) was introduced and this further improved on the timeliness, completeness and accuracy of reports. This was followed by training of health workers and support supervision at all levels to ensure correct use of the tools. However, despite of all these interventions data quality challenges still exist at health facility, district and national levels. Analysis of eHMIS immunization data between January and April 2015, showed that Kabarole district had inconsistent DPT3 coverage. The cause of this data discrepancy in the district was not known. We conducted this study to establish the source of immunization data quality inconsistency and suggest interventions.

## Methods

Kabarole District lies between 00 15” N and 10 00” N latitudes and 300 00” E 310 15” E Longitudes. It is Located in Western Uganda, about 300 km from the city center of Kampala. It has a total population of 469236, of which 3.3% (15330) are less than 1 year old [7]. This study was conducted between July and August 2016.

We adapted the immunization data quality audit, which is a standard World Health Organisation tool used to assess the robustness of national reports based on administrative monitoring systems [8]. Six senior health workers capable of working independently were selected and trained for 4 days. These were deployed for 10 days to all health centers (n = 49) within Kabarole District that conduct vaccination services. We interviewed health workers who handle and or manage vaccination services in these health centers.

To determine the consistence of the reporting system, we estimated the verification factor (VF). The VF was estimated by dividing the number of diptheria-pertussis-tetanus third dose (DPT3) vaccinations administered to children under one year of age during the audited year (2015) as recounted in health facility records filled at the time when children are vaccinated, by the annual DPT3 vaccinations reported in health facility reports found at the district health offices [8]. A VF of less than 100% suggested that the number of DPT3 recounted at the health facility was lower than that reported at district level (over-reporting); a VF of over 100% implied that all DPT3 recounted could not be traced back in the reports at district level (under-reporting). To characterize reporting consistency at the health facility level, results were put into three categories: consistent if the ratio (re-counted DPT3/reported DPT3) was ≥85% and ≤115%; over reported if ≤85%; under reported if the ratio was ≥115 [8].

We also assessed the timeliness, completeness, uniqueness and quality index of the administrative immunisation reporting system [9, 10].

Timeliness was determined by identifying the number of health unit reports that were submitted on or before the deadline (7^th^ of the following month) at the district health office.

Completeness was obtained by determining the number of health units which submitted their monthly reports to the district in a particular month compared to the maximum number of reports expected.

Uniqueness was assessed by identifying whether immunization data could be recorded more than once based on how it’s identified or how it appears. The question aimed as assessing whether there were any other tools used for tallying immunization data apart from HMIS Form 073a.

Quality Index (QI) was used to assess the quality of the data collection process. In calculating the QI score, 1 score was given for each question or observation that was correctly answered and zero score for any wrong answer. QI was computed by dividing the total number of correctly answered observations by the total number of answers and or observations for a particular component. The questions and observations in the QIs were grouped into 3 components: recording practices, storage and reporting practices, monitoring and evaluation. Denominators used at district and national level and systems design at national level were not assessed in this study.

### Data analysis

Descriptive statistics were used to summarize quantitative data. Categorical variables were described using frequency and percentages, while continuous variables were summarized using mean (range). Correlation analysis was used to determine the relationship between VF and QIs of health facilities.

### Ethical consideration

Approval was sought from Makerere University School of Public Health Institutional Review Board (SS 5000) and National Council for Science and Technology. Permission was also sought from the district authorities and management of the selected health facilities. Informed verbal consent was also sought from health workers after comprehensive explanation of the study and its objectives. The study did not pose any risk to the participants. Anonymity was guaranteed by not writing participants’ names and confidentiality was maintained by only researchers having access to information provided by the respondents.

## Results

**Fig 1:**
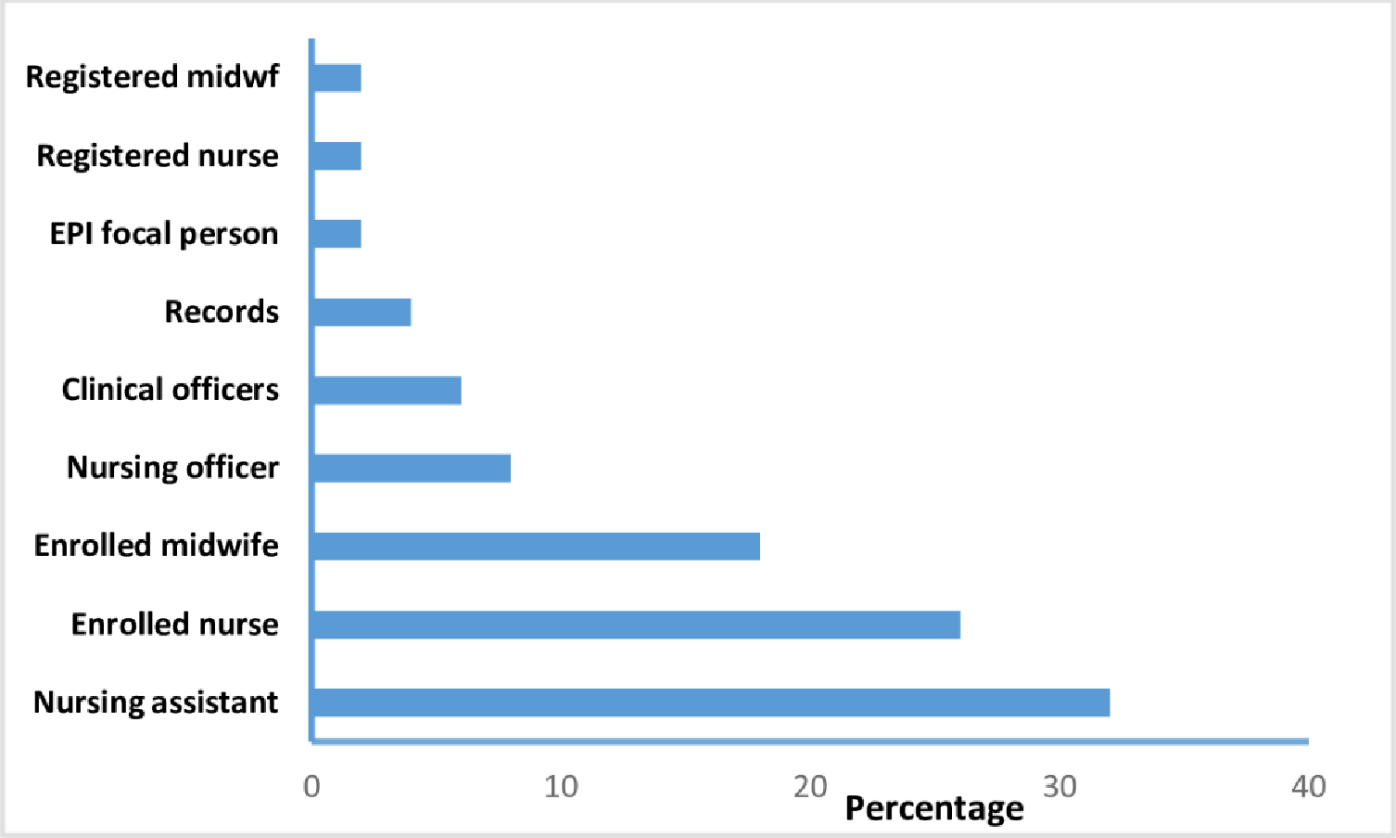
Graph showing percentage of respondents by category during 2015 immunization data quality audit, Kabarole District, Uganda.

The 2015 DQA in Kabarole District was done in all the 49 health centers that conduct vaccinations (Table 1). The Majority of our respondents were nursing assistants 32% (16/50) and enrolled nurses 26% (13/50) Fig 1.

**Table 1:** Percentage of health centers conducting vaccinations in Kabarole District, from 2015 immunization data quality audit.

Health center by level	Number of government health centers	Number of non governmental health centers	Total number of health centers	Number of health centers conducting vaccination	% of health center conducting vaccination
Regional referral hospital	1	0	1	1	100
Hospital	0	3	3	3	100
1V	3	3	6	3	50
111	24	7	31	31	100
11	21	12	33	11	36
**Total**	**49**	**25**	**74**	**49**	

Health center 11; located at parish level and serves around 5000 people. Health center 111; located at sub-county level and serves around 10000 people. Health center IV; located at county level and serves around 100,000. Hospital; located at district level and serves between 100000–1000000. Regional referral hospital; located at a region and serves 1000000–2000000 people.

### Timeliness and completeness of reporting

**Fig 2:**
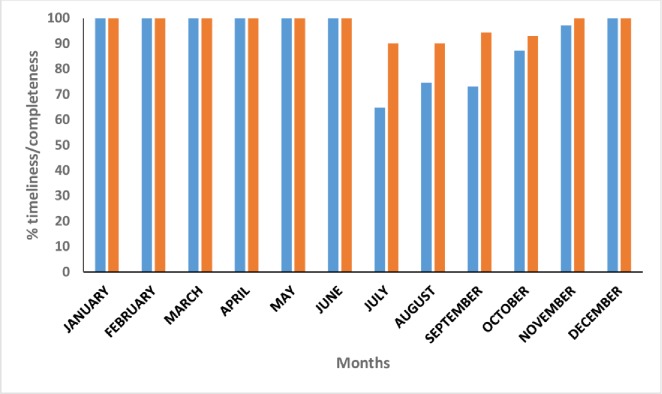
Timeliness and completeness of reporting from 2015 immunization data quality audit, Kabarole District, Uganda.

From January to June 2015, all health centers submitted their reports timely. Between July and November timeliness reduced but remained above 60%. Completeness also reduced during this period but remained above 90% (Fig 2). The dates for submission of reports to the District Health Officer (DHO) were known by most of the respondents interviewed 90% (45/50).The DHO’s office had a register to monitor timeliness and completeness of reporting.

### Consistence of immunization data

The mean health center verification factor was 87% (Table 2). Sixty five percent (32/49) of the health centers had consistent data, 27% (13/49) overreported and 4% (2/49) underreported.

**Table 2:** Verification factor by health center level, from immunization data quality audit 2015, Kabarole District, Uganda.

Health center level	Mean verification factor	Range
**Regional referral hospital**	**94.1**	**94.1–94.1**
**Hospital**	**96.9**	**94–101**
**1V**	**99.9**	**93–108**
**111**	**86.5**	**26–125**
**11**	**83.6**	**0.0–115**
**All health center levels**	**87**	**0.0–125**

(Fig 3). Verification factors for 2 health centers were not calculated because the incharge with the key for the records was away.

**Fig 3:**
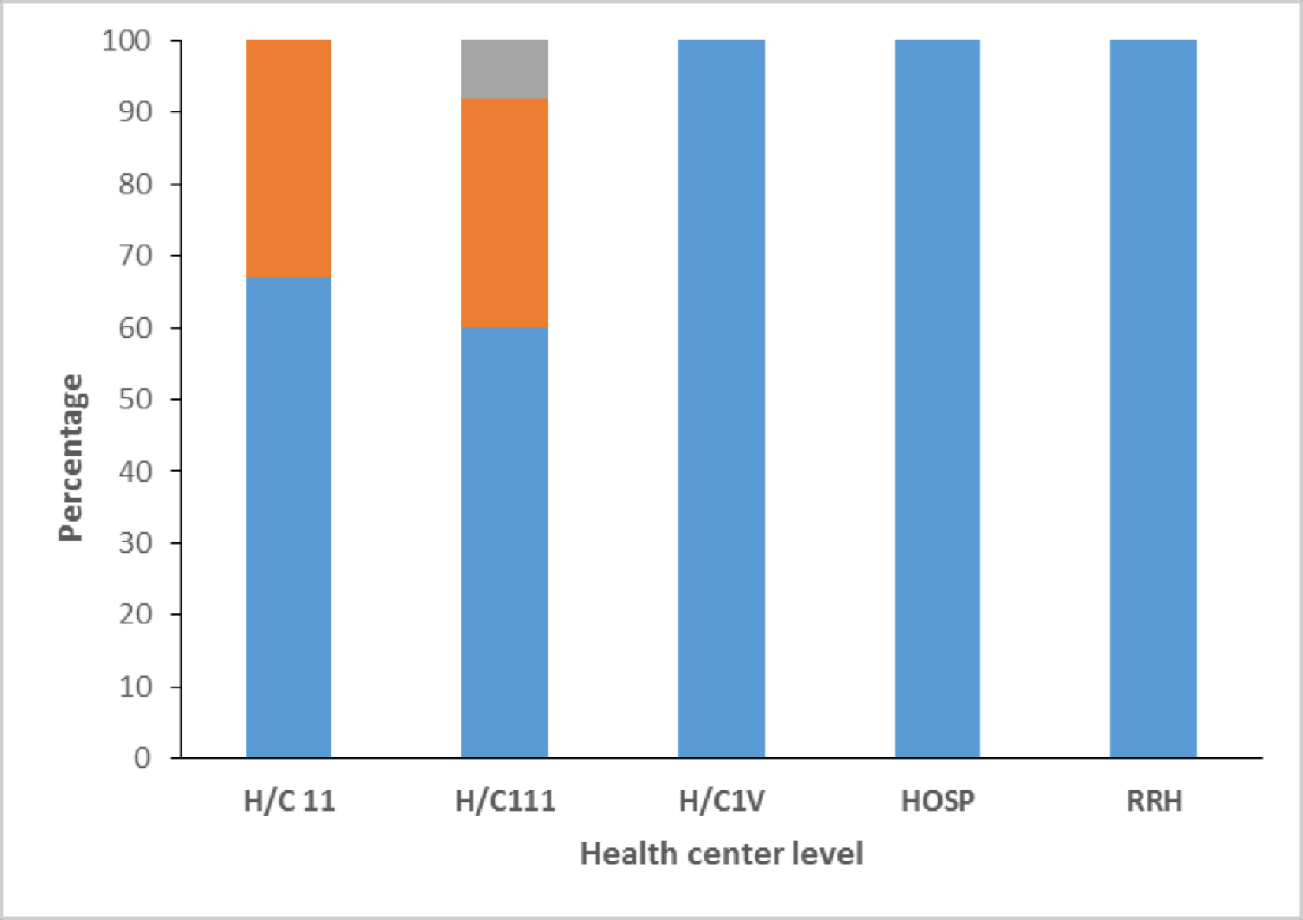
Consistency of DPT-3 vaccination by health center level, from immunization data quality audit, Kabarole district 2015.

The factors that affected immunization data quality under the data dimension included; arithmetic errors 20% (10/49) and uniqueness i.e. the health centers used other items to tally immunization data like exercise books and plain papers 53% (26/49).

Quality index scores varied at all levels of health service delivery. Mean QI for the 49 Health Centers that conduct immunization was 62% (Table 3).

**Table 3:** Mean quality indices for the different levels of healthcare delivery, from immunization data quality audit; Kabarole District, 2015.

Health center level	Mean QI (%)	Range
Regional referral hospital	62	13–100
Hospital	71	50–100
1V	82	64–92
111	70	18–100
11	51	27–92
All health center levels	62	13–100

The mean QIs for the 3 different componets assessed were; monitoring and evaluation 43%, recording 67%, and storing/reporting 76% (Table 4).

**Table 4:** Mean quality indices for the different components of the monitoring system in Kabarole District health centers, from immunization data quality audit, 2015.

Component	Mean QI (%)	Range
Monitoring and Evaluation	43	12.5–87.5
Recording	67	19–100
Storing/reporting	76	25–100

The factors that affected the data collection process under each of the components were: recording component; omission of tally sheet data from HMIS reports 14 (29%), irregular update of vaccine and injection material control book 11(22%); storing/reporting component; poor storage practices like lack of designated storage place 38 (78%), lack of files for keeping records 28 (57%), tally sheets not arranged in order 20 (41%), lack of access to records because incharge has moved with the key 2 (4%), missing tallysheets 13 (33%); monitoring and evaluation component; inability to classify target population according to immunization strategy 49 (100%), catchment area maps not displayed 30 (61%), graphs showing coverage and drop out rates not displayed 20 (41%), involvement of the community during planning rarely done 2(4%).

**Fig 4:**
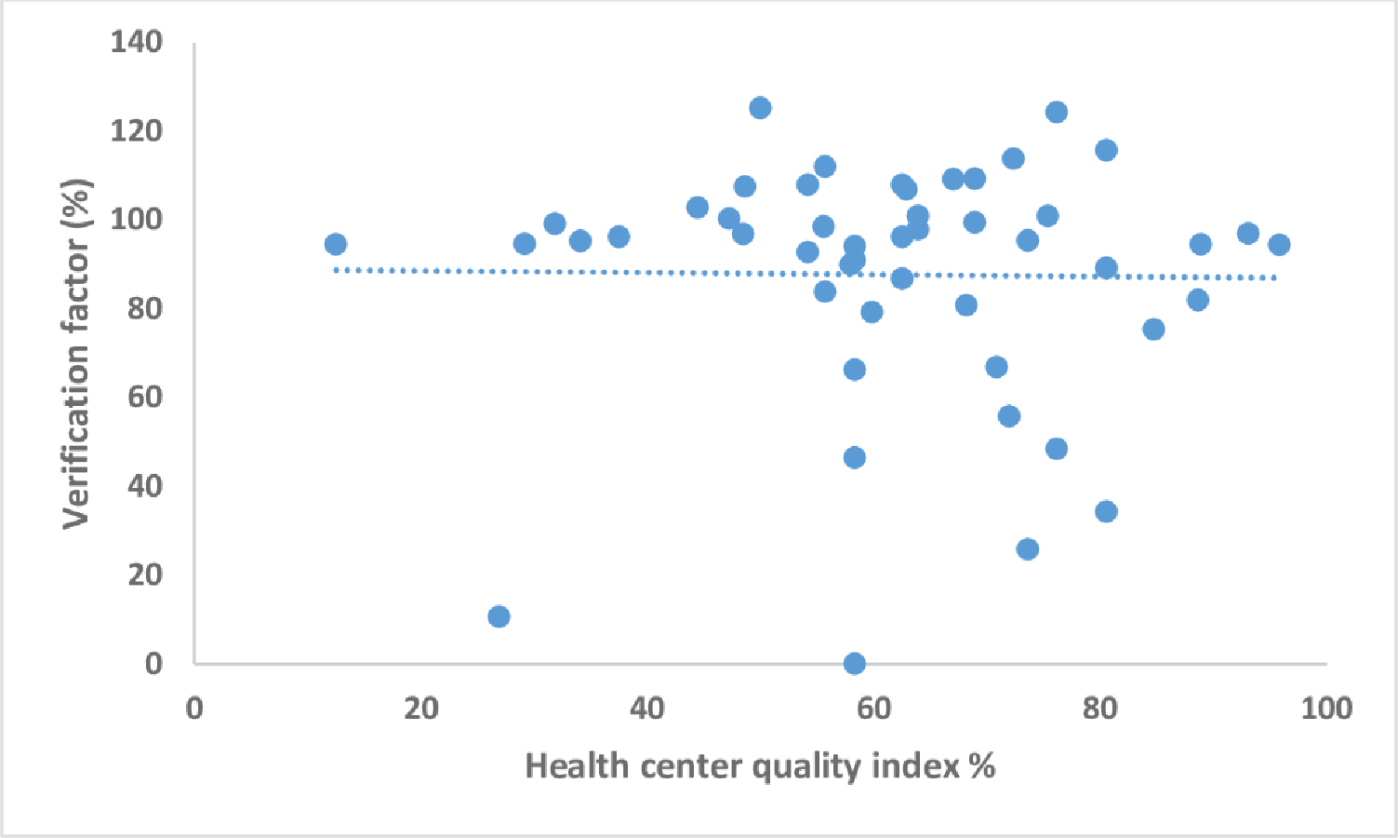
Correlation between verifaction factor and quality index score at health center level, in Kabalore District, from data quality audit 2015.

There was a weak positive correlation between the health center verification factor and quality index though this was not statistically significant (r = 0.014; p = 0.92) (Fig 4).This suggests that when the QI of the different health centers increase there is little or no change in the VF.

## Discussion

The immunization data quality audit conducted in Kabarole District in July 2016 showed that 27% (13/49) of the health centers overreported and 4% (2/49) underreported. The mean health center verification factor was 87%. The mean quality indices for the 3 different componets assessed were; monitoring and evaluation 43%, recording practices 66%, and storing/reporting 75%. The factors that influenced immunization data quality included; arithmetic errors, uniqueness, omission of tally sheet data from HMIS reports, irregular update of VIMCB, poor storage practices like lack of designated storage place, lack of files to keep records, poor arrangement of tally sheets, lack of access to records because incharge has moved with the key, missing tallysheets and inadequate data use.

This study highlights the quality of immunization reports from health centers about the number of children under 1 year, who have been vaccinated with all 3 doses of diptheria-pertusis-tetanus vaccine. DPT-3 is a key indicator that is used in the assessment of the effectiveness of an immunization service. The estimation of the verification factor in this study, underpins the consistency of the immunization data which district local governments and national ministry of health use for planning and decision making. A VF of < 100 showed that the audit team was not able to verify all the doses of DPT-3 reported to have been administered by the health workers within these facilities (overreporting). Also a VF of >100 showed that a large number of DPT-3 was recorded as having been administered at the health centers than were found at the district health office. Inconsistencies in data quality were noted in health center 11 and 111 which are located at parish and sub-county levels respectively. We also observed gross understaffing at these levels with inadequate knowledge and skills in data management. It’s however suprising that despite of all these factors, some of the health centers at these levels had good quality data that could be relied on for discission making. This implies that remedial actions need to be agreed upon by district local governments and ministry of health that focus on these levels of health service delivery, if immunization data quality is to improve. Underreporting of immunization data affects decision making at all levels of health service delivery. For instance, when a district underreports DPT-3, the national ministry of health may conduct periodicic intesfied routine immunization in that particular district and yet those funds could have been utilised much better elsewhere. On the contrally, when a district overreports, this may hinder projects aimed at scaling-up vaccination services. The likely causes of overreporting and underreporting under the data dimension were; arithmetic errors, uniqueness and omission of tally sheet data from HMIS reports. The findings from this study are similar to those from an immunization data quality audit conducted in 22 countries between 2002–2003, though with higher percentages of overreporting 40% (223/557) and underreporting 7% (38/557) in the different health facilities across countries [8]. Another study done in Manicaland province Zimbabwe in 2013, found similar findings though they purposively selected 21 health centers from the 7 districts [11]. Our study on the other hand, focused on all health facilities that conduct vaccination within the district.

Timeliness and completeness of reporting are the other key attributes under the data dimension that were assessed during this audit. Its important to note that these are among the commonly assessed data quality attributes [2, 8, 12]. During the audit year, all health centers submitted timely and complete reports between January and June. This was then followed by a decline in the level of reporting and timeliness between July and October before returning to normal in November and December. The audit however was not able to assess the factors that contributed to the decline in the level of reporting and timeliness during this period. Timely reporting of EPI data is essential in planning, monitoring, evaluation and subsequent interventions. This is well examplified when using the reach every district strategy that was developed by World Health Organization in 2002. This strategy requires that during review of the EPI data, different parishes, sub-couties and counties are categorised depending on DPT-1 coverage and DPT1 to DPT3 drop-out rate and then classified as either good or poorly performimg depending on access and utilization of vaccination services[13]. Late reporting of EPI data jeopardises this process and impides program performance. Several studies done have shown that delayed reporting negatively affects program perforamnce [8, 14]. The definition of completeness however, varied in several studies. A review of data quality assessment done, defined completeness as the number of facilities that submit reports to the district, similar to what we used in our study [15]. Other published studies defined completeness as the proportion of reports with all data elements filled [16, 17]. It’s therefore important to explicitly define the meaning of the variable so that data users are not confused during interpretension of findings from studies.

This DQA also revealed particular challenges in the data collection processes in the different health centers. The mean quality index score for the health centers that conduct immunization was 62%. Our findings showed wide variations in recording practices (mean QI = 66%), storage and reporting (mean QI = 75%) and monitoring/evaluation (mean QI = 43%). The quality of the systems index assisted us to estimate the overall quality of the immunization data collection processes.The monitoring and evaluation component was the worst performing and yet it’s the component responsible for providing timely feedback.The feedback given helps in collecting shortcomings noticed in the other components.The district local governments and ministry of health therefore, need to put mechanisms that foster timely feedback, if immunization data quality is to improve. Although these components were individually assessed, they don’t work in isolation, rather they work in synergy and weakness in any of the components affects the overall data quality. The district local governments and ministry of health therefore, need to address each of these components, if immunization data quality is to be improved. Similar studies have evaluated the quality of immunization monitoring system using quality indices [12, 18]. Though only three components were assessed in the data collection processes (recording, storage/reporting, monitoring and evaluation), a lot of useful information was generated to underpin the likely causes of data quality gaps.The factors that are likely to have influenced immunization data quality under the data collection process included; irregular update of VIMCB, poor storage practices due to lack of designated storage place, lack of files to keep records, tally sheets not arranged in order, lack of access to records because incharge has moved with the key and missing tallysheets. Studies done elsewhere have shown similar findings [19].

### Strength and limitations

The strength of this study lies in the fact that, all the health centres within the district that conduct immunization services were assessed. However, this study had several limitations; it focused on one district and therefore the findings cannot be generalizable to the whole country. Secondly only 3 components of the monitoring system were assessed instead of the recommended 5 components. Thirdly, the factors outlined as affecting data quality do not prove causality.

## Conclusion

This study showed that lower level health centers contributed significantly to the inconsistencies in immunization data. There were also wide variations between the quality indices of recording practices, storage/reporting, monitoring and evaluation. We recommended that District Local Governments and Ministry of Health strengthen the 3 components of the immunization reporting system at all levels and focus on improving data quality at lower levels of health service delivery.
